# Artificial Intelligence and Digital Technology in Cardiovascular Imaging: A Narrative Review

**DOI:** 10.3390/biotech15010022

**Published:** 2026-03-03

**Authors:** Constantinos H. Papadopoulos, Dimitris Karelas, Christina Floropoulou, Konstantina Tzavida, Dimitrios Oikonomidis, Athanasios Tasoulis, Evangelos Tatsis, Ioannis Kouloulias, Nikolaos P. E. Kadoglou

**Affiliations:** 1 Echocardiographic Laboratory, 2nd Cardiology Department, Korgialeneio-Benakeio Red Cross Hospital, 11526 Athens, Greece; papcost@gmail.com (C.H.P.);; 2 Medical School, University of Cyprus, 2029 Nicosia, Cyprus

**Keywords:** artificial intelligence, cardiovascular imaging, echocardiography, cardiac magnetic resonance, computed tomography, nuclear cardiology, machine learning, deep learning, radiomics, digital health

## Abstract

The rapid expansion of digital technologies and artificial intelligence (AI) has profoundly transformed cardiovascular imaging, enabling more precise, efficient, and reproducible assessment of cardiac structure and function. This narrative review summarizes recent advances in AI-driven methods across echocardiography, cardiac computed tomography, cardiac magnetic resonance, and nuclear imaging, with emphasis on image acquisition, automated quantification, and diagnostic and prognostic interpretation. We reviewed contemporary literature describing machine-learning and deep-learning applications for image reconstruction, segmentation, radiomics, and multimodal data integration. Current evidence demonstrates that AI improves image quality, reduces acquisition and analysis time, and enables automated, highly reproducible measurements of chamber volumes, function, tissue characterization, coronary anatomy, and myocardial perfusion, while facilitating advanced pattern recognition for differential diagnosis and risk stratification. Furthermore, digital platforms support remote acquisition, tele-echocardiography, and AI-assisted training of non-expert operators. Despite these advances, challenges remain regarding external validation, generalizability across vendors and populations, explainability, data governance, and regulatory compliance. In conclusion, AI and digital technologies are reshaping cardiovascular imaging by enhancing accuracy, efficiency, and accessibility, but their safe and effective clinical integration requires robust multicenter validation, transparent reporting, and ethical-legal frameworks that ensure trust, equity, and accountability.

## 1. Introduction

The exponential growth of computational power over the past two decades has fueled the so-called “Big Data Revolution,” enabling the storage and analysis of unprecedented volumes of digital information [1]. This transformation has laid the groundwork for the development of artificial intelligence (AI) and its integration into medical clinical practice, particularly within the field of cardiovascular imaging. In this context, imaging data are no longer viewed as isolated snapshots; rather, when integrated with clinical and laboratory parameters, they become the substrate for advanced diagnostic procedures leading to prognostic and therapeutic decision-making [2].

AI encompasses a broad range of computational models designed to mimic human cognition and generate autonomous, data-driven insights. Machine learning (ML) and deep learning (DL) have emerged as the two principal pillars of AI. ML algorithms utilize statistical methods to detect patterns and associations, enabling accurate predictions on novel datasets [3]. In the field of cardiovascular imaging, this facilitates the automated recognition of anatomical structures, classification of abnormalities, and precise quantification of parameters such as chamber volumes or left ventricular ejection fraction [4]. DL represents a more advanced paradigm, inspired by the architecture of human neural networks. By layering multiple interconnected nodes, DL systems capture highly complex, nonlinear relationships within imaging datasets [5]. This capability enables the development of robust diagnostic and prognostic models, for instance, those differentiating the several types of left ventricular hypertrophy or predicting time-to-event outcomes such as death or myocardial infarction, clustering data from perfusion images and clinical parameters [6]. Recent evidence also suggests that large language model–based artificial intelligence tools may further support cardiovascular practice by assisting clinicians in information retrieval, guideline interpretation, patient education, and clinical decision support. However, their reliability in complex clinical scenarios remains under evaluation [7].

Building on these foundations, this review explores the expanding role of AI in cardiovascular imaging, focusing on the recent advances in echocardiography, computed tomography (CT), cardiac magnetic resonance (CMR), and nuclear imaging, such as SPECT, alongside the integration of radiomic feature analysis for the detection and analysis of the underlying myocardial disease. Additionally, current challenges, such as clinical validation, generalizability, interpretability, and regulatory frameworks for AI, must be overcome to successfully translate AI-driven tools into routine clinical practice. The literature reviewed was identified through targeted searches of PubMed and Web of Science, focusing on studies published in English in the last two decades. Search terms included combinations of the following terms: “artificial intelligence,” “machine learning,” “deep learning,” “cardiovascular imaging,” “echocardiography,” “cardiac magnetic resonance,” and “computed tomography.” Relevant original articles, reviews, and consensus documents were selected based on their methodological quality, clinical relevance, and contribution to understanding current and emerging applications of AI in cardiovascular imaging.

## 2. Applications in Cardiovascular Imaging

The role of digital technology and AI in cardiovascular imaging extends beyond the evident enhancement of image quality (Table 1). Recent advances focus predominantly on the automation of image acquisition and quantification, and DL algorithms are now capable of providing real-time guidance to novice operators, ensuring the capture of diagnostic echocardiographic views [8,9].

Meanwhile, growing research efforts are directed toward automated image interpretation and prognostic modeling. For example, DL workflows now classify echocardiographic views and calculate several parameters such as LVEF without human input [10]. Furthermore, multimodal AI frameworks are being developed across CT, MRI, and nuclear modalities to provide comprehensive diagnostic decision support [11], with the ultimate objective of achieving seamless clinical integration. In parallel, digital innovations have transformed accessibility and training: AI-supported tele-echocardiography has introduced the remote control [11], and cloud-based DICOM/PACS platforms have made image sharing and interpretation globally accessible, as exemplified by the RSNA Image Share Project [12].

### 2.1. Image Quality

Modern digital and AI methods—particularly DL for reconstruction, denoising, motion correction, and super-resolution—can substantially enhance image quality across cardiovascular imaging modalities:

Echocardiography: Digital technology has led to an impressive improvement in spatial, temporal, and contrast resolution, enabling the optimal and highly detailed visualization of even the most challenging anatomical structures.

Cardiac CT: DL image reconstruction enables dose reductions of over 40% without compromising diagnostic accuracy, while super-resolution DL algorithms improve vessel edge sharpness, stent visualization, and increase the amount of agreement with findings from invasive angiography [13,14,15].

Cardiac MRI: DL cine reconstruction halves acquisition time while preserving volumetric accuracy and edge sharpness. Moreover, DL-based late-gadolinium enhancement improves scar conspicuity and contrast-to-noise ratios [16,17,18].

Nuclear cardiology: DL-based attenuation correction restores diagnostic performance of myocardial perfusion SPECT without CT, and DL denoising allows PET and SPECT imaging at lower counts or doses while preserving lesion detectability and quantitative accuracy [19,20,21].

**Figure 1:**
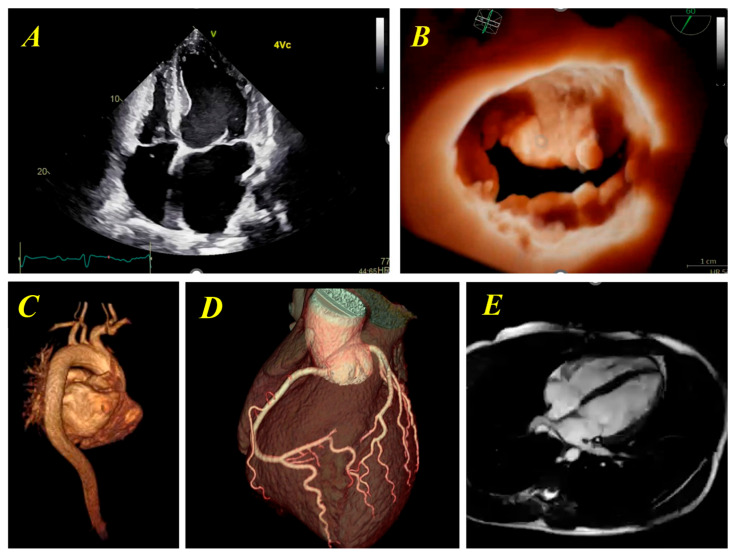
Representative examples of cardiovascular imaging modalities illustrating the breadth of anatomical and functional information available across techniques. (**A**) Apical four-chamber echocardiographic view demonstrating excellent delineation of endocardial borders. (**B**) Three-dimensional echocardiographic reconstruction of the mitral valve from a left ventricular perspective, clearly showing the leaflets, scallops of the posterior leaflet, and chordal insertions. (**C**) Three-dimensional computed tomography reconstruction illustrating the dimensions and the course of the aorta. (**D**) Three-dimensional reconstruction of coronary computed tomography angiography allowing the assessment of potential congenital defects or stenoses. (**E**) Cardiac magnetic resonance four-chamber view with clear distinction of cardiac chambers.

Collectively, these innovations improve resolution, contrast, and spatiotemporal fidelity, while reducing noise, radiation dose, and acquisition time. They enable cardiovascular imaging to be more accurate, faster, safer, and more reproducible, with direct benefits for downstream interpretation and clinical decision-making (Figure 1).

The images presented are illustrative examples of routinely acquired cardiovascular imaging datasets and are not intended as direct comparisons between standard and AI-supported image acquisition. In the examples shown, AI applications primarily relate to post-processing, automated segmentation, quantification, and visualization rather than enhancement of raw image quality during acquisition.

Although AI has demonstrated potential for improving image quality through advanced reconstruction, motion correction, and artifact reduction, quantitative evaluation of these improvements—particularly in suboptimal imaging conditions—remains heterogeneous across modalities and algorithms. As this is a narrative review, AI-related image quality enhancement is discussed conceptually rather than through head-to-head comparisons with standard acquisition methods, since objective and standardized metrics were not consistently reported across the available literature.

Differences in apparent image quality across figures, therefore, reflect variations in acquisition conditions, patient characteristics, imaging modality, and the specific stage of the imaging workflow at which AI is applied.

### 2.2. Automation

Automation represents one of the most transformative applications of AI in contemporary cardiovascular imaging. By leveraging semi-automated or fully automated acquisition and quantification, AI systems can recognize, segment, and measure cardiac structures with minimal operator intervention, thereby significantly enhancing efficiency and workflow. A recent study demonstrated a ~25% reduction in scan time and over 50% fewer console keystrokes using an AI-assisted multiplane imaging protocol, while maintaining image quality and interpretability [22]. Moreover, AI-driven processing facilitates rapid, accurate analysis, slashing echocardiographic measurement and report creation time by approximately 70% compared to manual methods [23].

Advanced algorithms applied to standard imaging projections have consistently demonstrated excellent agreement and reproducibility compared to manual measurements by expert operators, provided image quality is adequate and anatomical complexity is limited. For instance, a formal validation study of a DL–enabled automated echocardiographic workflow involving 23 parameters showed that the discrepancy between DL and human experts was actually lower than the inter-observer variability among three core-lab readers [24]. As a result, AI significantly narrows the proficiency gap between novice and senior imaging specialists, enhancing diagnostic precision and laboratory throughput.

Automation in cardiovascular imaging spans numerous critical domains, ranging from standardized image acquisition and automated chamber quantification to lesion characterization, functional assessment, and integration into multimodal diagnostic pipelines. For instance, convolutional neural network–based pipelines have shown high accuracy in view classification, segmentation, and calculation of functional metrics (e.g., volumes, strain) from 2D echocardiography data [25]. Similarly, AI has been successfully leveraged to automate chamber volumetry from non-contrast gated CT scans, achieving near-perfect correlation coefficients (r = 0.98 for LV and 0.95 for LA volumes) compared to gold-standard references [26]. Furthermore, reviews in interventional imaging highlight how AI enables the fusion of CT, MRI, echocardiography, and nuclear data into unified diagnostic workflows to comply with the principles of precision medicine [2].

LVEF AI-based fully automated three-dimensional echocardiography using HeartModelAI demonstrates excellent inter- and intra-observer reproducibility for left ventricular volumes and ejection fraction, with strong correlations compared to cardiac magnetic resonance imaging (r = 0.918 for LV end-systolic and r = 0.911 for LV end-diastolic volume indices) [27]. Although LV volumes were systematically underestimated and ejection fraction overestimated, manual contour correction improved the accuracy of LVEF assessment, supporting the value of AI-assisted quantification for reproducible longitudinal evaluation. Moreover, 3D echocardiography provides superior reproducibility for serial LVEF assessment compared with 2D techniques, which is particularly important in cardio-oncology, where clinical decisions depend on small temporal changes in ejection fractions. In patients undergoing chemotherapy, non-contrast 3D echocardiography demonstrated the lowest temporal variability, with minimal detectable LVEF changes of approximately 5–6%, compared with about 10–13% using 2D methods, reducing the risk of misclassifying measurement variability as cardiotoxicity [28]. Three-dimensional echocardiography is recommended for serial assessment of left ventricular function in patients undergoing cancer therapy because of its superior reproducibility compared with two-dimensional techniques. Non-contrast 3D echocardiography demonstrates lower temporal variability for LVEF measurement (approximately 5.6%) than 2D echocardiography (about 9.8%), supporting its use for detecting clinically meaningful changes during cardio-oncology surveillance [29].

Collectively, these developments accelerate clinical decision-making and support more equitable access to high-quality cardiovascular imaging (Table 2).

**Table 2:** Automation in cardiovascular imaging with artificial intelligence.

Modality	Parameters Automatically or Semi-Automatically Assessed by AI
Echocardiography (Figure 2, Figure 3, Figure 4 and Figure 5)	Chamber dimensions, wall thickness, volumes, and systolic/diastolic function (global and regional), using conventional, 3D, or speckle-tracking techniques Valve morphology with automated identification of leaflet segments Parameters for grading valvular stenosis and regurgitation
Cardiac Magnetic Resonance (CMR) (Figure 6)	Myocardial mass, volumes, and ejection fraction Tissue characterization (presence of edema/inflammation, extracellular volume expansion, fibrosis) via T1/T2 mapping, relaxation times, and mapping techniques with or without contrast
Cardiac Computed Tomography (CT) (Figure 7)	Coronary Artery Calcium Score Degree of luminal stenosis and fractional flow reserve estimation (FFR-CT) Quantitative and qualitative plaque analysis, with accurate recognition of high-risk features such as low-attenuation plaque and positive remodeling Higher accuracy compared to manual measurements or clinical risk scores
Nuclear Cardiology (Figure 8)	Detection and quantification of myocardial perfusion defects
Intravascular Imaging	Plaque characterization and classification (morphology, type, burden) Estimation of stenosis severity with greater accuracy in identifying vulnerable plaque features

**Figure 2:**
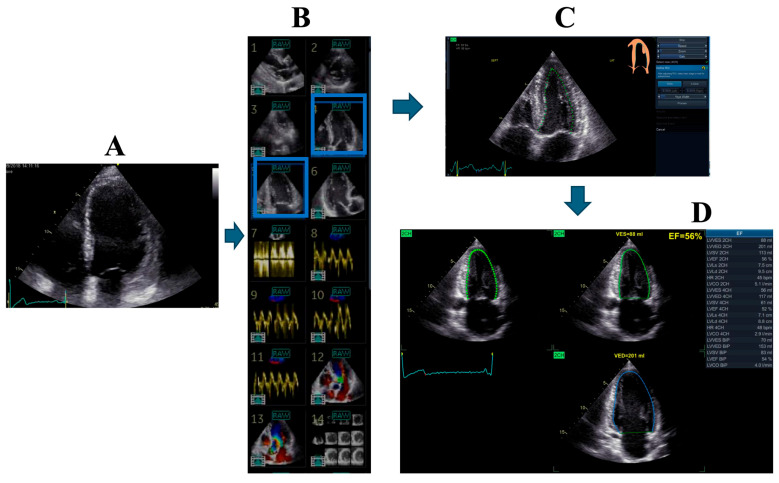
Example of AI-assisted echocardiographic analysis. AI-based algorithms are applied for automated segmentation and quantification of cardiac chambers from routinely acquired echocardiographic images. In this example, AI support is used to facilitate measurements and reduce operator dependency, rather than to enhance image quality at the acquisition stage. (**A**) Manual selection of a four- (or two-) chamber view image by the operator. (**B**) Automated retrieval by the software of the optimal corresponding cardiac cycle and frame rate from the stored study images (highlighted in blue). Panels 1–6 show 2D echocardiographic views; 7–11 spectral and tissue Doppler; 12–13 color Doppler; and 14 serial short-axis views derived from the apical long-axis. (**C**) Automatic endocardial border delineation in both diastole and systole for the selected views. (**D**) Automated calculation of LV volumes and function, including ejection fraction, stroke volume, and cardiac output.

**Figure 3:**
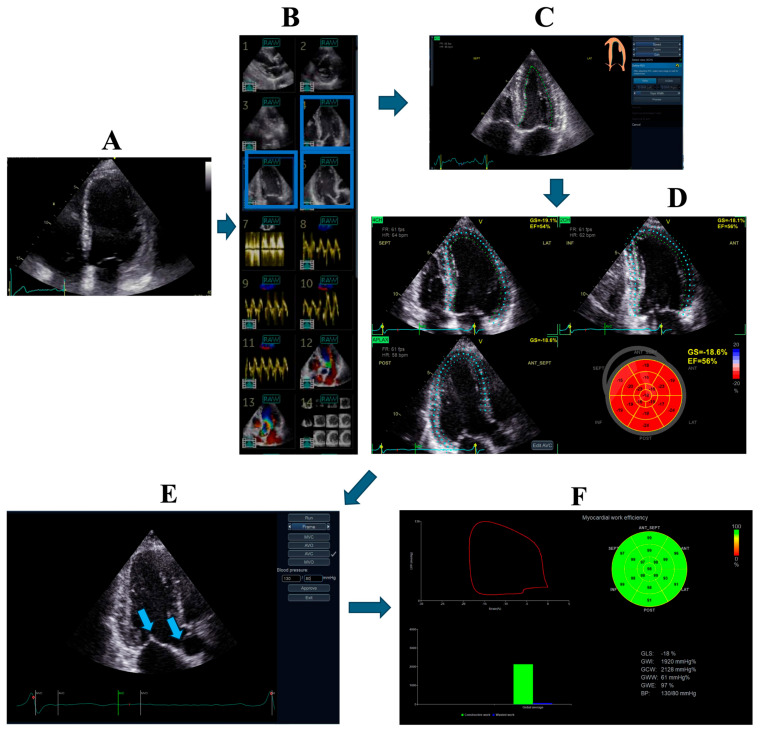
Example of automated calculation of left ventricular myocardial strain and myocardial work using the two-dimensional viewing in echocardiography. (**A**) Manual selection of a four-chamber view by the operator. (**B**) Automatic retrieval of the additional optimal apical views (two- and three-chamber) from the stored dataset (matching cardiac cycle and frame rate requirements, highlighted in blue). Panels 1–6 show 2D echocardiographic views; 7–11 spectral and tissue Doppler; 12–13 color Doppler; and 14 serial short-axis views derived from the apical long-axis. (**C**) Automated endocardial and epicardial border delineation in systole and diastole across all views. (**D**) Automated quantification of global and segmental strain with graphical dis-play. At steps C and D, the operator can edit and correct the measurements if needed. (**E**) Input of brachial arterial blood pressure and automated or manual timing of aortic and mitral valve open-ing/closure. Arrows indicate aortic and mitral valves. (**F**) Display of strain-derived pressure–volume loops and myocardial work indices (global constructive work, wasted work, global work efficiency).

**Figure 4:**
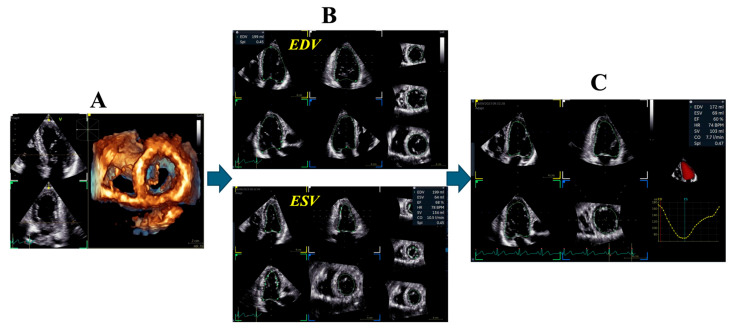
Example of automated calculation of left ventricular (LV) volume and ejection fraction using the three-dimensional viewing in echocardiography. (**A**) Manual selection of a pyramidal apical four-chamber acquisition by the operator. (**B**) Identification of two landmarks (the apex and the center of the mitral leaflet coaptation), followed by automated endocardial border delineation in systole and diastole across multiple reconstructed planes. (**C**) Automated quantification of left ventricular volumes and function (ejection fraction, stroke volume, cardiac output). At this step, the operator has the option to edit and refine the contours.

**Figure 5:**
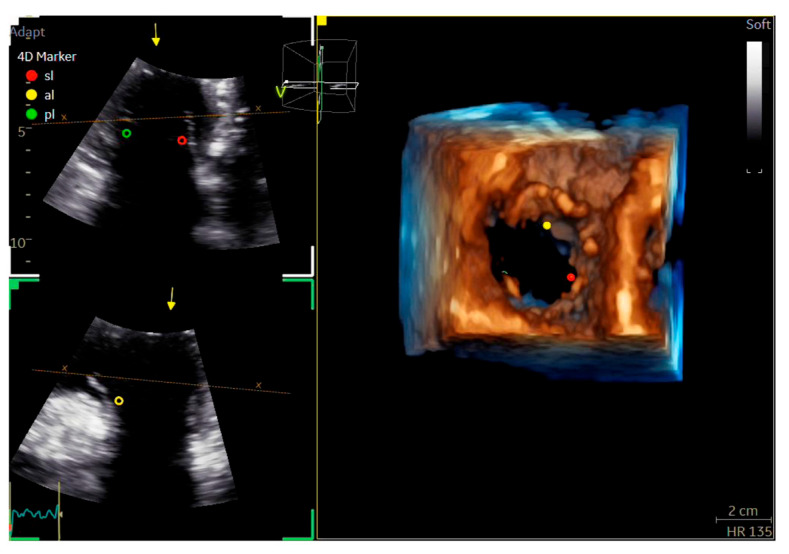
Example of automated identification of the tricuspid valve leaflets in three-dimensional viewing in echocardiography.

**Figure 6:**
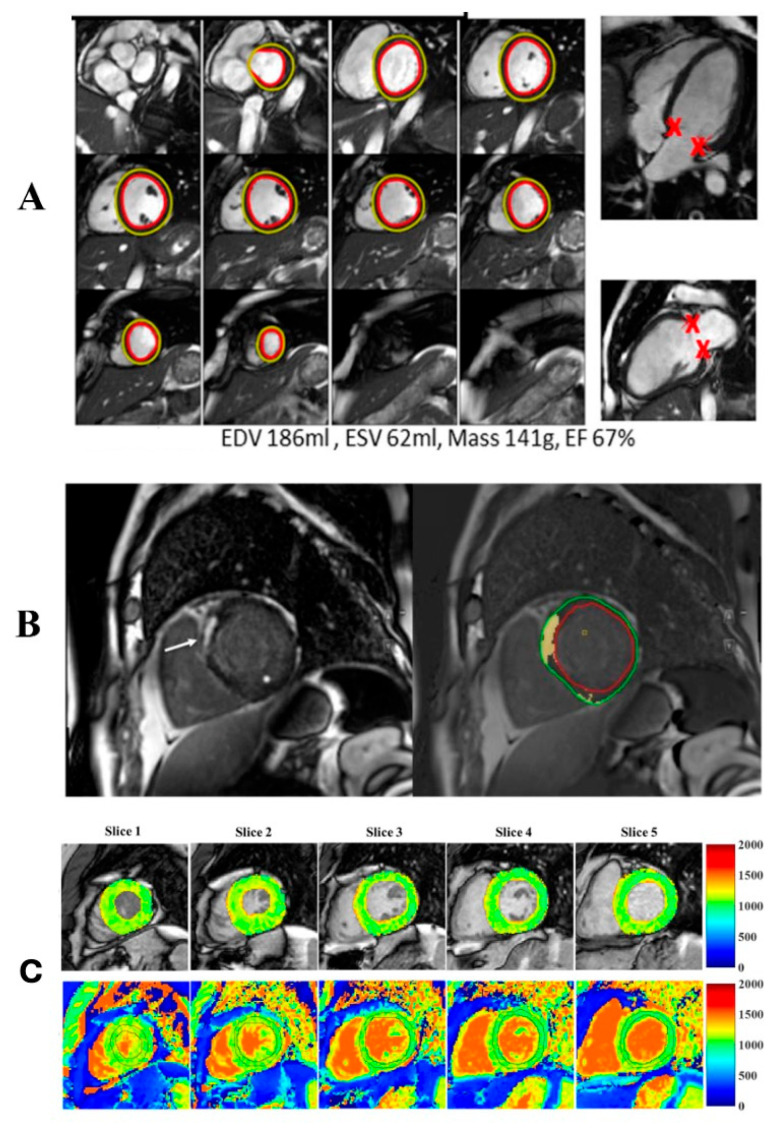
Artificial intelligence applications in cardiac magnetic resonance imaging. AI-based methods are used for automated segmentation, functional quantification, and tissue characterization from routinely acquired CMR datasets. In this figure, AI does not aim to improve raw image quality but to enable efficient and reproducible analysis. (**A**) Automated delineation of the endocardium (red) and epicardium (yellow) with automated calculation of left ventricular mass, volumes, and ejection fraction. ‘X’ denotes the annotated mitral annular reference point. This approach reduces processing time from approximately 10 min to less than 1 min. Adapted from Davies RH et al. [30]. (**B**) Quantitative assessment of myocardial fibrosis (late gadolinium enhancement—LGE) in a patient with myocardial infarction. Left: visual depiction of LGE (arrow). Right: automated quantification of fibrosis extent (yellow) in relation to total myocardial mass (between green and red contours). Adapted from Lanzafame LRM et al. [31]. (**C**) Automated (top row) versus manual (bottom row) quantification of T1 mapping, showing excellent agreement (1091 ± 59 ms vs. 1089 ± 59 ms, respectively). Adapted from Fahmy AS et al., [32].

**Figure 7:**
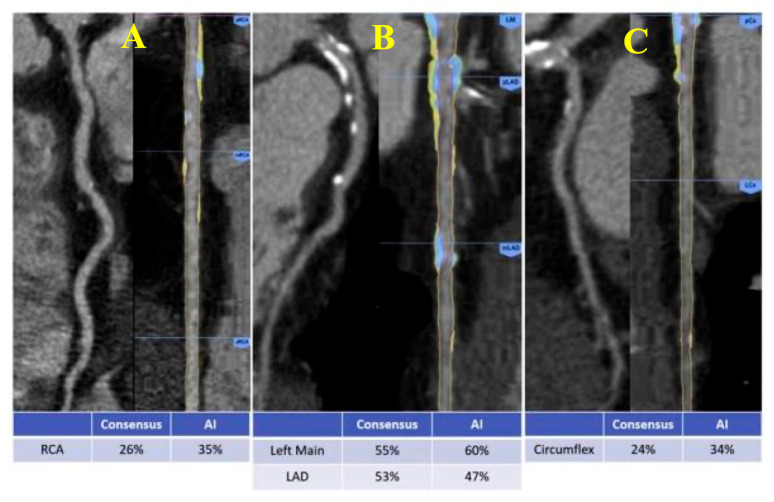
Automation in Coronary Computed Tomography Angiography (CCTA). Comparison of stenosis assessment performed by an expert Level-3 reader versus AI-based automated analysis across major coronary arteries: (**A**) Right coronary artery (RCA); (**B**) Left main (LM) and Left Anterior Descending Artery (LAD); (**C**) Left Circumflex Artery (LCx). Adapted from Choi AD et al. [33].

**Figure 8:**
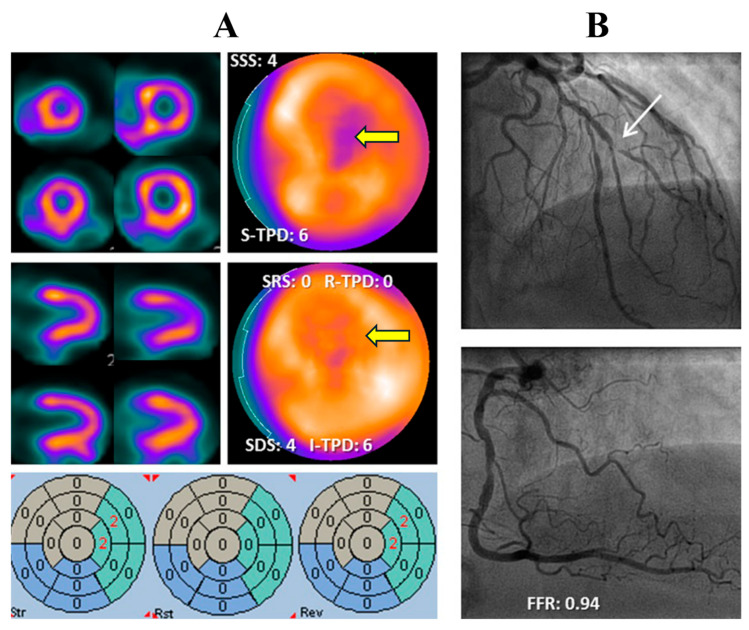
Automation in Myocardial Perfusion Imaging (MPI). Stress ((**A**), top row) and rest ((**A**), middle row) single-photon emission computed tomography (SPECT) images demonstrating a reversible perfusion defect in the mid-to-apical anterolateral wall (yellow arrows). Automated quantification maps ((**A**), bottom row) highlight perfusion deficits using a standardized 17-segment model. (**B**). Invasive coronary angiography showing subtotal stenosis of a diagonal branch (white arrow) with corresponding physiological assessment (FFR: 0.94). Adapted from Driessen RS et al. [34].

At stages C and D, the operator retains the option to edit and correct measurements if required. Total analysis time is less than one minute, compared with 2–3 min using the conventional manual method.

The process requires approximately 2–3 min compared with >5 min using the conventional manual method for strain analysis alone.

The entire process requires approximately 1–2 min, compared with the longer duration of conventional manual methods.

After placing dedicated markers on the two-dimensional images to indicate the leaflet positions, the software automatically recognizes the septal (SL, red), anterior (AL, yellow), and posterior (PL, green) leaflets in the 3D dataset. This automated process requires less than one minute and provides high accuracy in assessing leaflet anatomy and number, even in the presence of anatomical variations.

### 2.3. Interpretation of Images (Diagnosis and Prognosis)

In terms of image interpretation, ML models are increasingly being developed based on features of normal and pathological cardiac anatomy and physiology. These models can reliably differentiate between normal cardiac function and various disease states, as well as among distinct cardiac pathologies—for instance, by distinguishing ischemic cardiomyopathy from non-ischemic dilated cardiomyopathy with high accuracy via echocardiographic ML algorithms [35]. Moreover, by integrating ML and DL techniques into imaging findings and clinical variables, predictive models are being developed to predict patient outcomes and treatment response; A notable example is the ML-based analysis of SPECT imaging, combined with demographic data. It has been shown to provide incremental prognostic value in patients with coronary artery disease, surpassing traditional clinical methods [35].

Radiomics represents a cutting-edge analytical discipline that converts standard medical images into high-dimensional quantitative datasets. By extracting a vast array of features, including texture, shape, intensity gradients, and voxel interrelationships from radiological studies, radiomics enables the detection of subtle imaging phenotypes far beyond the detection threshold of human visual assessment [36]. In cardiovascular imaging, this emerging field has shown promise across multiple modalities. For example, radiomic analysis of coronary plaques on CCTA has been used to identify vulnerable lesions by detecting microstructural heterogeneity and subtle remodeling features that traditional imaging typically overlooks [37]. Similarly, myocardial radiomics applied to CMR has facilitated differentiation of acute myocarditis from other inflammatory conditions by analyzing textural patterns on T2 maps, achieving excellent sensitivity and specificity (>89% and >92%, respectively) [36].

Beyond diagnostics, radiomics also enhances prognostic modeling. A recent myocardial radiomics model derived from cine CMR demonstrated strong predictive power in dilated cardiomyopathy, while CT-based models improved outcome prediction in chronic infarction settings [38,39]. These quantitative imaging signatures can be integrated into ML pipelines, enabling precise risk stratification and personalized therapy selection.

However, translating these findings into clinical practice requires the resolution of substantial issues—including the standardization of acquisition protocols, cross-vendor reproducibility, and the conduction of robust external validation studies. The importance of high-quality, multicenter datasets and consensus-driven feature extraction is well recognized—as illustrated by the Image Biomarker Standardization Initiative’s (IBSI) efforts toward feature calculation harmonization and the successful reduction in multicenter radiomic variability with harmonization techniques (e.g., ComBat and histogram matching) in cardiovascular MRI studies [40,41].

While artificial intelligence holds significant promise for enhancing image analysis, workflow efficiency, and decision support in cardiovascular imaging, current AI-based tools should not be regarded as fully autonomous or universally reliable systems. Most algorithms are developed and validated within specific datasets and clinical environments, raising concerns regarding generalizability, potential overfitting, and performance variability when applied to external populations or suboptimal imaging conditions. In addition, issues related to model explainability and transparency remain relevant, particularly in complex clinical decision-making scenarios.

Importantly, direct comparative studies evaluating AI-assisted workflows against standard clinical practice are still relatively limited, and the available evidence is often heterogeneous across imaging modalities and applications. Moreover, the majority of commercially available or investigational AI algorithms are implemented as fixed, static models corresponding to a specific stage of development, without clearly established mechanisms for continuous learning or adaptive updating in real-world clinical settings. These limitations underscore the need for ongoing validation, regulatory oversight, and prospective studies to define the safe and effective role of AI in routine cardiovascular imaging practice.

The main active research areas for AI applications in diagnostic and prognostic imaging are summarized in Table 3.

### 2.4. Education

Modern digital systems have introduced transformative avenues for education in cardiovascular imaging. One significant application is training non-experts in imaging techniques: dedicated educational software powered by ML and DL algorithms can now provide real-time guidance to non-experts—such as nurses or technicians—during echocardiographic examinations. These platforms offer continuous probe-positioning cues until diagnostically acceptable views are achieved and automatically stored. An example is the FDA-cleared HeartFocus platform (by DESKi), which enabled novices to acquire echocardiograms of expert quality in a comparative study of over 200 patients [44].

Similarly, ultrasound-guidance technologies, such as Caption Guidance and UltraSight, have received FDA clearance. These AI-enabled tools assist users in real time through graphical feedback and positioning cues to capture standard 2D transthoracic echocardiography (TTE) views with minimal training [8,9].

Beyond software-based guidance, immersive simulation has become a vital and indispensable component of modern curricula. High-resolution Virtual Reality (VR) simulators, constructed from authentic human cardiac scans, provide trainees with high-fidelity anatomical models for hands-on learning, eliminating patient risk during the initial learning curve [8].

**Figure 9:**
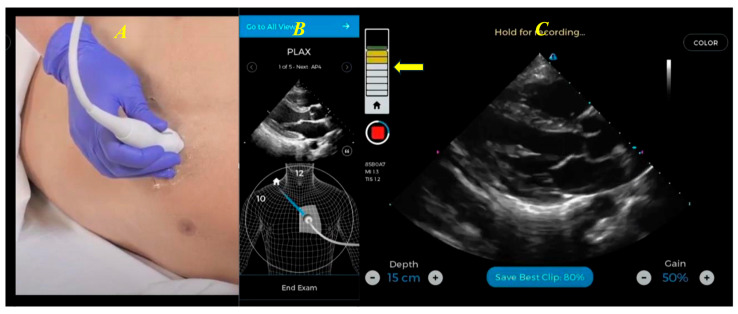
Training software for image acquisition, designed for cardiologists or non-imaging personnel (e.g., nurses, technicians). The transducer is positioned on the patient’s chest (**A**), while the software provides real-time guidance (instructions for movement, adjustment, or rotation of the probe) to the operator (**B**), in order to obtain the optimal parasternal long-axis view. The software uses a feedback bar (yellow arrow) where green indicates optimal positioning, and once achieved, the clip is automatically stored (**C**). Modified from Narang et al. [8].

**Figure 10:**
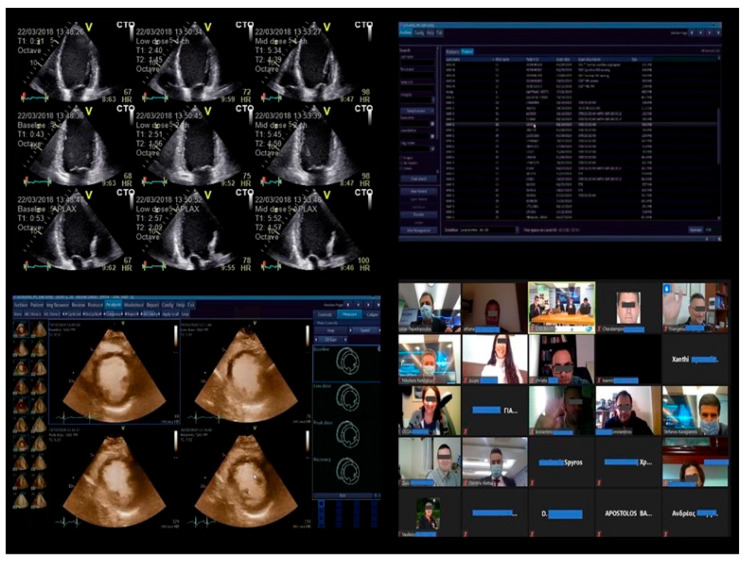
Remote training seminars in cardiovascular imaging. Training sessions can be conducted remotely, allowing instructors and trainees to participate from anywhere in the world. Through dedicated software, the instructor can access the trainee’s workstation, provide real-time guidance, and correct potential errors.

These training tools effectively complement seminar-style education (Figure 9 and Figure 10). Simulator-based programs—utilizing either virtual reality or high-fidelity mannequins—offer standardized, repeatable environments for both transthoracic and transesophageal echo training. Although live-patient training remains essential, randomized trials show that simulators significantly improve theoretical understanding and technical skills, especially for complex imaging protocols [45].

## 3. Limitations/Ethical–Legal Issues

Despite rapid technological progress, the clinical deployment of AI in cardiovascular imaging remains constrained by limited generalizability, dataset shift, and insufficient external validation: many models developed on single-center or vendor-specific datasets often underperform when faced with variations in scanner types, imaging protocols, or patient demographics. This underscores the imperative for rigorous multicenter validation and calibration prior to clinical adoption. Recent cardiology evaluations emphasize that variance across institutions and populations can introduce bias and degrade performance at the point of care, and call for prospective, human-benchmarked studies with outcome-relevant endpoints [46,47]. In parallel, significant deficits in transparency and reproducibility persist; updated reporting standards in medical imaging (CLAIM) and trial reporting guidelines (SPIRIT-AI/CONSORT-AI) mandate clear documentation of datasets, reference standards, preprocessing, model versioning, and human oversight to mitigate these risks and to enable replication [48,49].

Beyond technical accuracy, ethical and legal considerations are paramount: algorithmic bias can exacerbate health inequities; limited explainability complicates clinician trust and accountability; and large-scale data use raises privacy, consent, and governance challenges [46]. Legally, the EU Artificial Intelligence Act classifies most clinical imaging AI as “high-risk,” imposing obligations for risk management, high-quality data, human oversight, technical documentation, transparency, and post-market monitoring; mapping work across Europe highlights the breadth of binding policies that healthcare AI must satisfy alongside existing MDR/IVDR pathways [50,51].

To bridge these gaps, consensus frameworks such as FUTURE-AI translate these requirements into actionable principles: Fairness, Universality, Traceability, Usability, Robustness, and Explainability. Such frameworks provide the necessary governance scaffold for the safe, equitable, and accountable integration of AI into cardiovascular imaging [47].

The limitations and ethical considerations related to the use of AI in cardiovascular imaging are summarized in Table 4.

## 4. Future Perspectives

AI in cardiovascular imaging is rapidly advancing toward seamless clinical integration, propelled by the synergistic fusion of multimodal data. By synthesizing imaging findings with electronic health records (EHRs), genomics, and longitudinal data from wearable devices, AI enables a holistic approach to risk assessment and precision-tailored therapeutics [52]. Advances in explainable AI (XAI) are expected to improve clinicians’ trust in AI systems by enhancing the interpretability and transparency of model outputs [52]. In parallel, federated learning offers a scalable solution for multi-institutional model development without direct data sharing, thereby mitigating privacy concerns and enhancing interoperability. A simulated federated learning study using CMR data across four institutions achieved performance comparable to centralized training and demonstrated increased robustness to domain differences [53].

## Figures and Tables

**Table 1:** Role of digital technology and artificial intelligence in cardiovascular imaging.

Domain	Role of Digital Technology and AI
Image quality	Improved spatial, temporal, and contrast resolution, and noise reduction through advanced reconstruction techniques
Automation	Semi-automated and fully automated image acquisition, segmentation, and quantification; reduced operator dependency
Interpretation and Diagnosis	AI-driven recognition of patterns and anatomical structures; support in differential diagnosis and disease classification
Prognostic Modeling	Clustering of imaging and clinical data for treatment guidance and outcomes prediction
Accessibility and Sharing	Enhanced storage, retrieval, and transmission of imaging studies through digital platforms
Training and Education	AI-enabled tools for physician training, standardization of imaging protocols, and remote learning

**Table 3:** Major active research areas in diagnosis and prognosis with AI in cardiovascular imaging.

Modality	Diagnosis/Differential Diagnosis	Prognosis
Echocardiography	Subtypes of heart failure with preserved ejection fraction (HFpEF) Types of myocardial hypertrophy (physiological, hypertrophic cardiomyopathy, amyloidosis, etc.) Constrictive pericarditis vs. restrictive cardiomyopathy	Sudden cardiac death risk prediction in cardiomyopathy
Cardiac MRI	Types of myocardial hypertrophy Myocardial viability LV/LA function Extent and patterns of late gadolinium enhancement (LGE) T1 and T2 mapping for tissue characterization Phase-contrast MRI for cardiovascular flow quantification [42,43] 4D flow MRI for comprehensive hemodynamic assessment	Risk stratification based on LGE burden and distribution Prognostic value of myocardial mapping parameters Hemodynamic biomarkers derived from phase-contrast and 4D flow MRI
Cardiac CT	Assessment of coronary artery stenosis severity	Prognosis in coronary artery disease (CAD) − Low attenuation and positive remodeling of atherosclerotic plaque − Plaque volume combined with the degree of stenosis − Perivascular adipose tissue analysis
Nuclear Cardiology	Diagnosis/detection/prognosis of obstructive CAD Myocardial viability	

**Table 4:** Limitations and Ethical–Legal Issues in the Use of AI in Cardiovascular Imaging.

Limitations	Ethical/Legal Issues
Reduced accuracy of automated measurements for quantification of cardiac chamber and valve function (moderate/poor image quality and complex anatomy). Limited availability of multicenter validation studies in diagnosis, quality of care, cost reduction, and patient prognosis.	Redefinition of the medical and paramedical staff roles. Data privacy concerns. The “black-box” problem: difficulty in interpreting results and measurements generated automatically by AI.

## Data Availability

The original contributions presented in this study are included in the article. Further inquiries can be directed to the corresponding author.
